# Liver fibrosis among infants with t(1;22)(p13;q13) acute megakaryoblastic leukemia: a case report and literature review

**DOI:** 10.3389/fonc.2024.1441318

**Published:** 2024-08-30

**Authors:** Nira Arad-Cohen, Ori Attias, Yaniv Zohar, Yoav H. Messinger

**Affiliations:** ^1^ Pediatric Hematology-Oncology Department, Ruth Rappaport Children’s Hospital, Rambam Health Care Campus, Haifa, Israel; ^2^ Pediatric Intensive Care Unit, Ruth Children’s Hospital, Rambam Health Care Campus, Haifa, Israel; ^3^ Rappaport Faculty of Medicine, Technion-Israel Institute of Technology, Haifa, Israel; ^4^ Department of Pathology, Rambam Health Care Campus, Haifa, Israel; ^5^ Cancer and Blood Disorders, Pediatric Hematology/Oncology Department, Children's Minnesota, Minneapolis, MN, United States

**Keywords:** acute megakaryoblastic leukemia, non-Down, liver fibrosis, pediatric, t(1;22)(p13;q13) translocation

## Abstract

This case report describes a 2-month-old girl with acute megakaryoblastic leukemia (AMKL) harboring the t(1;22)(p13;q13) translocation, resulting in the RBM15::MRTFA fusion gene. She presented with massive hepatosplenomegaly and liver fibrosis and achieved complete remission with chemotherapy; the liver fibrosis resolved within 2.5 months. After 12 years of follow-up, the patient remained in good health, without relapse. Reviewing the literature on eight additional similar cases of liver fibrosis, this subtype of AMKL predominantly affects female patients below 3 months of age, with a median onset at 6 weeks. High rates of severe complications were observed, with five of nine patients dying within 10 weeks of diagnosis. The authors hypothesized that the proliferation of abnormal megakaryoblasts within the liver leads to the release of profibrotic cytokines, such as TGF-β1, which induces liver fibrosis similar to that observed in transient abnormal myelopoiesis in Down syndrome. Careful monitoring of liver functions and reduced-intensity chemotherapy are recommended for this very young patient population. Nonetheless, long-term survival can be achieved with aggressive supportive care and treatment.

## Introduction

Acute megakaryoblastic leukemia (AMKL), categorized as subtype M7 of acute myeloid leukemia (AML) in the FAB classification, involves the abnormal proliferation of megakaryoblasts, accounting for only 1% of AML in adults, whereas in children, it accounts for between 4% and 15% of AML patients (1). In children, AMKL is divided into two groups: AMKL with Down syndrome (DS-AMKL) and another in children without Down syndrome (non-DS-AMKL). Within non-DS-AMKL, t(1;22)(p13;q13), resulting in RBM15::MRTFA (previously termed MKL1) fusion, has exclusively been observed in infants diagnosed with AMKL and is the most common genetic alteration in pediatric non-DS-AMKL (2, 3). It is characterized by organomegaly, myelofibrosis, and fibrosis affecting other organs (4).

We report a case of t(1;22)(p13;q13) AMKL presenting with massive hepatosplenomegaly and liver fibrosis that received chemotherapy, in which biopsy showed resolution of liver fibrosis and long-term (12 years) survival. We provide a literature review of nine patients, noting the early death of five of the nine patients and multiple complications in others. We suggest that hepatic fibrosis may be secondary to the proliferation of abnormal megakaryoblasts in the livers of these very young infants, akin to liver fibrosis noted in neonates with transient abnormal myelopoiesis (TAM) of Down syndrome (DS).

## Case description

A 2-month-old girl was referred after 2 weeks of vomiting and abdominal distention. The baby was the first child of this family, and the perinatal period was uneventful. On admission, she appeared sick and pale with no dysmorphism, mild dyspnea, and a markedly distended abdomen. The size of the liver and spleen could not be evaluated due to abdominal distension. The white blood cell count (WBC) was 30,000/µL, hemoglobin (Hb) was 10.1 g/dL, platelets were 36,000/µL, and circulating blasts were 12%. Liver functions showed ALT = 244 U/L (30–65), AST = 350 U/L (5–40), bilirubin total = 0.75 mg/dL, LDH = 1,900 U/L (60–225), albumin = 2.7 g/dL, and coagulation studies were normal. Abdominal ultrasonography (US) showed an enlarged liver (9 cm), non-homogenous echogenicity of the liver with lobulated borders suspected of infiltration, and splenomegaly (10 cm) with a large amount of ascites.

Bone marrow aspiration (BMA) showed that 47% of the marrow nucleated cells were blasts with blebs. Flow cytometry revealed positive myeloid markers (CD33, CD34, and CD117) and CD41. Cytogenetic evaluation revealed t(1;22)(p13;q13) with no other chromosomal aberrations, and FLT3-ITD was negative, confirming the diagnosis of AMKL. The patient did not have CNS involvement. Treatment was initiated according to the AML-BFM 98 protocol. Owing to a massively distended abdomen, she developed respiratory failure and was intubated and ventilated. A large amount of ascites resulted in abdominal compartment syndrome, requiring peritoneal drainage draining 500–1,000 cc of transudate daily, with no blasts in the peritoneal fluid.

**Figure 1:**
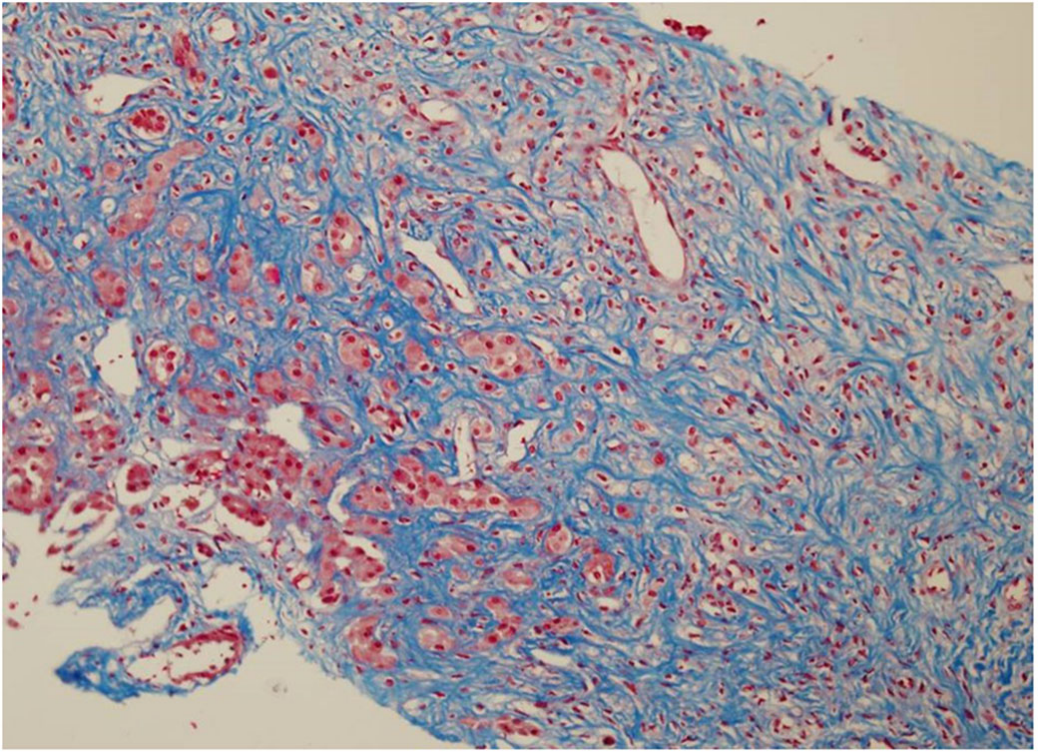
First liver biopsy (day 15 after diagnosis): massive fibrosis replacing most of the liver tissue with no inflammation and no signs of leukemia (Masson’s trichrome, 20X).

On day 15, the bone marrow was severely hypocellular with no blasts. By that time, the liver enzymes had completely normalized, but there was no decrease in the liver size. The viral profile was negative. A liver biopsy was performed to help guide future chemotherapy and showed massive fibrosis replacing most of the liver tissue with no inflammation or signs of leukemia (**Figure 1**). On day 28, scheduled chemotherapy was not administered because of ongoing complications including intubation, ventilation, massive hepatomegaly, severe hypoalbuminemia, peritoneal drainage, upper GI bleeding requiring gastroscopy, and recurrent bacteremia. The BMA was hypocellular, with no blasts and a normal karyotype.

**Figure 2:**
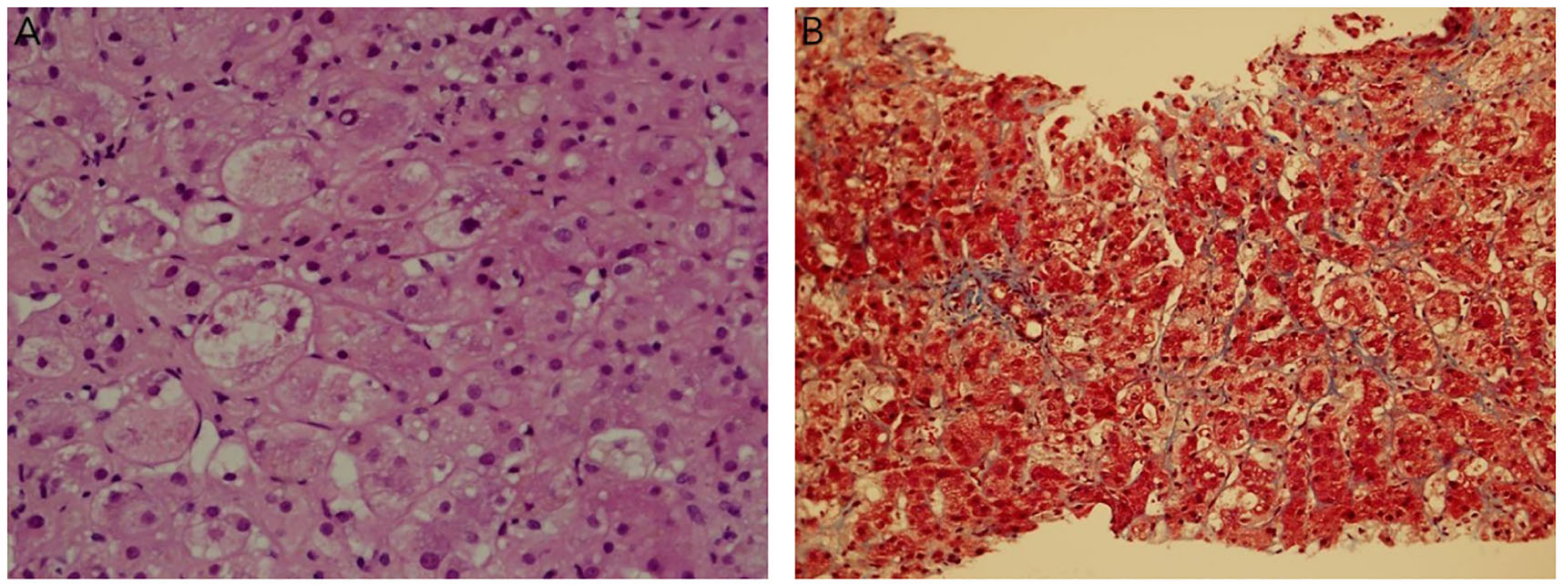
Repeat liver biopsy (3 months after diagnosis). **(A)**. Focal ballooning degeneration of hepatocytes, bile deposits, no inflammation or leukemic infiltrates (H&E 40X). **(B)**. Minimal sinusoidal fibrosis (Masson’s trichrome, 20X).

At 3 months post-diagnosis, she was clinically better and ventilated with CPAP, with increased WBC and bone marrow complete morphological and cytogenetic remission. However, she developed cholestatic jaundice, with a bilirubin level of up to 24 mg/dL. Abdominal US showed signs of cirrhosis, portal hypertension with many collaterals, and splenomegaly (12.4 cm). Viral studies were again negative. A repeat liver biopsy revealed extensive ballooning degeneration of the hepatocytes (**Figure 2A**), bile deposits, no inflammation or leukemic infiltrates, and minimal fibrosis (**Figure 2B**), indicating drug-induced cholestasis. The cholestatic jaundice was attributed to antibiotics and prolonged total parenteral nutrition (TPN).

At 4 months after the diagnosis, the patient underwent a tracheostomy and received oxygen, and the bilirubin level started to decline. Since the BMA showed 2% blasts, she received two courses of intermediate-dose cytarabine 500 mg/m^2^ for 4 days, with no anthracyclines due to her liver status. After two courses, the bone marrow showed complete morphological, cytogenetic, and molecular remission, with a normal number of megakaryocytes. She developed hypersplenism-induced refractory thrombocytopenia with marked splenomegaly (12–14 cm), portal hypertension, and grade 2 gastric varices. Splenectomy was performed without complications, resulting in the recovery of platelet counts and the resumption of chemotherapy. The patient underwent the complete AML-BFM98 protocol; however, the high-dose cytarabine–mitoxantrone (HAM) course was omitted. However, maintenance therapy was abbreviated after seven courses of cytarabine when the patient was 1 year and 5 months old because of recurrent bacterial infections and uncertainty regarding the efficacy of maintenance therapy in children with AML.

US evaluation at 4 years of age showed no evidence of portal hypertension. At 12 years of follow-up after the completion of chemotherapy, the patient remained in good health with no evidence of recurrent AMKL.

## Literature review

**Table 1** presents a literature review of nine cases of AMKL with t(1;22)(p13;q13) with liver fibrosis, including our patient. All patients were ≤3 months old (median, 6 weeks; range, 0–3 months) and were almost exclusively female (eight of nine). All patients presented with hepatomegaly; seven of nine had abdominal distension, two of nine had ascites, and one had liver failure. All had thrombocytopenia; most had anemia (average Hgb 8.4, range: 5.7–10.9 g/dL) and normal WBC count (average 24,500 × 10^9^/uL, range: 8,000–42,500); and six of nine had circulating blasts. The reported liver function was not severely elevated (except for the patient who presented with liver failure). When reported, the bone marrow showed marked myelofibrosis, which is common in AMKL. Cytogenetics noted t(1;22)(p13;q13) in eight of nine, and the other patient had insertion of part of the p13q31 of chromosome 1 on der(22) chromosome 1 into 22, resulting in the same translocation (7). Most comprehensive liver pathology is reported by Chan et al. (9), noting that: “several histologic patterns with megakaryoblasts in clusters in hepatic sinusoids, varying degrees of surrounding fibrosis and other areas with prominent fibrosis and effacement of the normal architecture. Aggregates of blasts, isolated bile ducts, and small islands of hepatic tissue could be seen amid the dense fibrous tissue. The megakaryoblasts also might be seen in large aggregates in portal veins, lymphatics, and central veins resembling carcinomatosis”. Notably, in two of the nine cases, no pathology of the liver was reported; in one case, fibrosis was suggested by us, demonstrating liver elasticity >10 times normal (6); and in the other case, computed tomography (CT) showed prominent hepatomegaly with patchy fibrotic areas (8). Combination AML therapy or low-dose cytarabine was administered to eight of the nine, and one patient died within 2 days of diagnosis before the initiation of chemotherapy. Of the four surviving patients, complete remission was achieved within 4–7 weeks. Overall, major complications were reported in eight of the nine cases, including sepsis, multiorgan failure, ascites requiring drainage, liver failure, veno-occlusive disease (VOD), coagulopathy, and massive subarachnoid hemorrhage. There were four survivors at a follow-up range of 9 months to 12 years, but five of the nine patients (56%) died within 2 days to 10 weeks after diagnosis.

**Table 1:** Clinical data of nine infants diagnosed with t(1;22)(p13;q13) acute megakaryoblastic leukemia and liver fibrosis.

Case number	Gender	Age (month)	Presentation	WBC (x10^9^/L)	Plt (x10^9^/L)	Peripheral Blasts (%)	Liver biopsy	AML treatment	Outcome	Ref.
1	M	1	Abdominal distension, ascites	8	16	0	Diagnosis—megakaryoblasts and fibrosis	Yes	No CR, died of MOF within weeks	Lewis et al. (5)
2	F	1	Abdominal distention, vomiting, hepatosplenomegaly	25	23	3	Autopsy after 2nd induction—extensive liver fibrosis without blasts	Yes	No CR, died of MOF after 10 weeks	Feng et al. (6)
3	F	3	Gastrointestinal bleeding, hepatosplenomegaly	28	31	49	ND, US—liver elasticity of >10 times the UNL, suggestive of hepatic fibrosis	Yes^a^	CR for 3 years	Feng et al. (6)
4	F	3	Abdominal distension, hepatosplenomegaly	36	86	2	Diagnosis—neoplastic cells, dense collagen fibrosis Day 28 biopsy-dense fibrous tissue, no blasts	Yes	CR for 2.5 years	Margolskee et al. (7)
5	F	1	Liver failure, marked abdominal distension, hepatomegaly, and ascites	43	18	1.5	ND, CT—prominent hepatomegaly with patchy fibrotic areas	Low-dose cytarabine	Died due to severe hepatic failure after 6 weeks	Nakano et al. (8)
6	F	1.5	Abdominal distension, hepatosplenomegaly	21	140	0	Postmortem—2 weeks after 1st induction—fibrosis and blasts	Yes	Died due to a massive subarachnoid hemorrhage after 2 weeks	Chan et al. (9)
7	F	2	Hepatosplenomegaly	15	80	0	Diagnosis—marked fibrosis and blasts	Yes	CR for 9 months	Chan et al. (9)
8	F	0	Bleeding, abdominal distension, hepatosplenomegaly	16	30	10	Diagnosis—extensive fibrosis and scattered blasts	Untreated	Died after 2 days	Chan et al. (9)
9	F	2	Abdominal distention, ascites, hepatosplenomegaly	30	36	12	2 weeks after 1st induction—massive fibrosis, no blasts After 3 months of diagnosis—ballooning degeneration of hepatocytes, minimal fibrosis, no blasts	Yes	CR for 12 years	Current case

a Addition of antifibrosis therapy (retinoin and α-tocopheryl acetate).

CR, complete response; CT, computed tomography; MOF, multi-organ failure; ND, not done; Plt, platelet; UNL, upper normal value; US, ultrasound; WBC, white blood cell.

## Discussion

The RBM15::MRTFA fusion gene, associated with t(1;22)(p13;q13), was identified in 13.7%–20% of pediatric AMKL cases (3, 8), with median age at diagnosis of 0.33–0.7 years [median reported by Bernstein (4) — 0.33 years, Inaba (10) — 0.55 years, Chisholm (3) — 0.61 years, and de Rooij (11) — 0.7 years]. The characteristic features of this leukemia include myelofibrosis and fibrosis in various organs (4, 5). Here we describe a patient with severe liver fibrosis resulting in severe portal hypertension and secondary hypersplenism inducing refractory thrombocytopenia, in whom fibrosis essentially resolved within 2.5 months. Follow-up ultrasonography revealed no portal hypertension after 4 years and long-term survival (12 years), with no relapse.

The reason why these nine patients with AMKL with t(1;22)(p13;q13) developed hepatic fibrosis is not known. The process of hepatic fibrosis may be similar to that described in Down syndrome TAM. In both cases, the patients were neonates or extremely young, and there was an abnormal proliferation of megakaryoblasts in the liver. Interestingly, in 1992, Miyauchi et al. suggested a hypothesis for hepatic fibrosis in TAM based on the myelofibrosis noted in AMKL (12). They suggested that similar to AMKL myelofibrosis, abnormal blasts in TAM proliferate in the liver during the fetal period, producing excessive cytokines, including TGF-β1, PDGF, and PF4, which stimulate growth and collagen synthesis in fibroblasts to cause liver fibrosis (12). We hypothesized that liver fibrosis in AMKL with t(1;22)(p13;q13) is akin to that in Down syndrome TAM, thus closing the circle of knowledge (from AMKL myelofibrosis → TAM liver fibrosis → AMKL liver fibrosis). In TAM, hepatic fibrosis is associated with elevated levels of cytokines, including IL-7, IL-8 (CXCL8), CCL2, and TGF-β1, and platelet-derived growth factors (PDGFα and PDGFβ) (13–17). A direct candidate for liver fibrosis is TGF-β1, which is secreted by megakaryoblasts and induces liver fibrosis (18). Additionally, the key cytokine, IL-8 (CXCL8), which is produced by liver cells, resident hepatic macrophages, and T cells, attracts neutrophils to release reactive oxygen species, causing local necrosis that results in fibrosis (14). In fact, exchange transfusion has been suggested to reduce abnormally elevated cytokines and liver fibrosis in TAM (17). At this time, TGF-β1 or IL-8 (CXCL8) levels were not measured in the cases noted in **Table 1**, including our case, but should be considered in the future.

We hypothesized that the reversal of fibrosis documented in our case may have been a result of the elimination of abnormal megakaryoblasts from the liver by chemotherapy, possibly eliminating the excretion of TGF-β1, allowing the regeneration of the liver with almost complete elimination of fibrosis (**Figure 2B**). The reversible hepatic fibrosis documented in our case was similar to that observed in TAM. Whether anti-fibrotic agents added to chemotherapy are necessary to reverse hepatic fibrosis is not clear since they were used with chemotherapy in one case (6) but were not used for the other three survivors.

RBM15::MRTFA AMKL is more prevalent in female patients than in male patients [female/male ratio, 3:1 (9) or 2.5:1 (3)]. Notably, the cases of RBM15::MRTFA with liver fibrosis seemed to occur almost exclusively in female patients (eight of nine cases). The median age of the subgroup with liver fibrosis was 6 weeks (**Table 1**), which was lower than the median age of 4–8 months reported in larger groups of RBM15::MRTFA AMKL (3, 4, 10, 11).

Although the outcome of RBM15::MRTFA is reportedly similar to that of other patients with childhood AML (3, 8, 10), we noted a poor outcome with early death in five of nine (56%) patients with liver fibrosis (**Table 1**). Inaba et al. (10) reported that of the AMKL patients who died early, balanced t(1;22) was the most common. Moreover, we noted high severe complication rates, which probably explains the particularly poor outcome in this very young subgroup (**Table 1**). Should these patients undergo early exchange transfusion to decrease the cytokines responsible for liver fibrosis as is suggested for some TAM (17) remains an open question since it was not performed in any of the cases described in **Table 1**.

Therapy for this subset of patients is extremely challenging owing to their significant comorbidities and young age. Although low-dose cytarabine was administered in some cases, it was probably insufficient to induce prolonged remission. AML-type combination chemotherapy was administered to all surviving patients. Additionally, cessation of chemotherapy due to severe complications is required for long periods, as in our case, suggesting that chemotherapy-free intervals do not result in rapid disease recrudescence.

We note that the cause of early deaths in the cases discussed in **Table 1** was not leukemia relapse but rather sepsis or multi-organ failure. Importantly, our patient survived emergent splenectomy, leading to the resolution of refractory thrombocytopenia, suggesting that drastic measures are possible in such cases. Finally, at this time, none of the surviving patients had AMKL relapse, although we noted a short follow-up of three cases (0.75, 2.25, and 3 years), and only our case was disease-free at the 12-year follow-up.

In conclusion, we describe a case of acute megakaryoblastic leukemia (AMKL) with RBM15::MRTFA fusion associated with severe hepatic fibrosis and portal hypertension that resolved with chemotherapy. We reviewed nine cases of RBM15::MRTFA AMKL with liver fibrosis, noting that this condition predominantly affects female infants and is associated with generally poor outcomes. The very young age of these patients, coupled with severe comorbidities, contributed to poor outcomes rather than leukemia relapse. It is crucial to monitor liver functions as liver failure (8) and veno-occlusive disease (7) have been observed in these cases. Furthermore, the chemotherapy dosages for these patients should be reduced as recommended for very young infants and neonates with AML (19). Finally, patients may tolerate extreme interventions, such as splenectomy, which may facilitate long-term survival for at least up to 12 years.

## Data Availability

The original contributions presented in the study are included in the article material. Further inquiries can be directed to the corresponding author.
